# Stemness and chemotherapeutic drug resistance induced by EIF5A2 overexpression in esophageal squamous cell carcinoma

**DOI:** 10.18632/oncotarget.4581

**Published:** 2015-07-20

**Authors:** Hong Yang, Xiao-dong Li, Ying Zhou, Xiaojiao Ban, Ting-ting Zeng, Lei Li, Bao-zhu Zhang, Jingping Yun, Dan Xie, Xin-Yuan Guan, Yan Li

**Affiliations:** ^1^ Sun Yat-sen University Cancer Center, State key Laboratory of Oncology in South China, Collaborative Innovation Center for Cancer Medicine, Guangzhou, China; ^2^ Guangdong Esophageal Cancer Institute, Guangzhou, China; ^3^ Department of Clinical Oncology, The University of Hong Kong, Hong Kong, China

**Keywords:** esophageal squamous cell carcinoma (ESCC), EIF5A2, stemness, chemoresistance

## Abstract

Esophageal squamous cell carcinoma (ESCC) is one of the most lethal malignancies of the digestive tract in East Asian countries. Multimodal therapies, including adjuvant chemotherapy and neo-adjuvant chemotherapy, have become more often used for patients with advanced ESCC. However, the chemotherapy effect is often limited by patients' drug resistance. This study demonstrated that EIF5A2 (eukaryotic translation initiation factor 5A2) overexpression induced stemness and chemoresistance in ESCC cells. We showed that EIF5A2 overexpression in ESCC cells resulted in increased chemoresistance to 5-fluorouracil (5-FU), docetaxel and taxol. In contrast, shRNAs suppressing *eIF5A2* increased tumor sensitivity to these chemotherapeutic drugs. In addition, EIF5A2 overexpression was correlated with a poorer overall survival in patients with ESCC who underwent taxane-based chemotherapy after esophagectomy (*P* < 0.05). Based on these results, we suggest that EIF5A2 could be a predictive biomarker for selecting appropriate chemo-treatment for ESCC patients and EIF5A2 inhibitors might be considered as combination therapy to enhance chemosensitivity in patients with ESCC.

## INTRODUCTION

Esophageal squamous cell carcinoma (ESCC) is the seventh most common cancer and the fifth most common cause of cancer-related death in the world [1]. Esophagectomy remains the mainstay of treatment for resectable disease. However, the prognosis for patients treated by surgery alone is poor. Recent developments in postoperative management, including chemotherapy and fractionized radiation therapy, have contributed to improvements in the surgical outcome [2–4]. Because chemotherapy is useful as adjuvant therapy before or after surgery for potentially resectable tumors, it has led to considerable interest in the identification of predictive biomarkers for selecting appropriate treatments for patients with ESCC.

Our previous study demonstrated that chromosomal amplification and hypoxia could induce the expression of EIF5A2, the eukaryotic translation initiation factor 5A2, and that it was important in epithelial-mesenchymal transition (EMT) and metastasis of ESCC [5]. Recently, the ablation or inhibition of EIF5A2 was also reported to enhance the chemosensitivity of HCC (hepatocellular carcinoma) cells to 5-fluorouracil (5-FU)/doxorubicin and breast cancer cells and bladder cancer cells to doxorubicin [6–9]. Despite these reports, the underlying mechanism of acquired chemosensitivity of silencing EIF5A2 is poorly defined. Because chemotherapy is becoming more widely used as adjuvant therapy for patients with ESCC, a better understanding of the mechanisms of chemoresistance of EIF5A2 is therefore critical for selecting treatments and the development of potential strategies to overcome chemoresistance.

In this study, we sought to explore the role of EIF5A2 in the chemoresistance of ESCC cells, and we tested the chemosensitivity in both EIF5A2 overexpressed cells and knock-down cells. In addition, EIF5A2 overexpression enhanced the stemness of ESCC cells and inhibited the apoptosis activated by chemotherapy treatment. Moreover, our results show that the EIF5A2 expression in resected ESCC specimens is correlated with a worse clinical outcome and response to adjuvant chemotherapy, suggesting an important clinical application of EIF5A2 in selecting appropriate chemo-treatments for patients with ESCC.

## RESULTS

### EIF5A2 increased the stemness of ESCC cells

**Figure 1:**
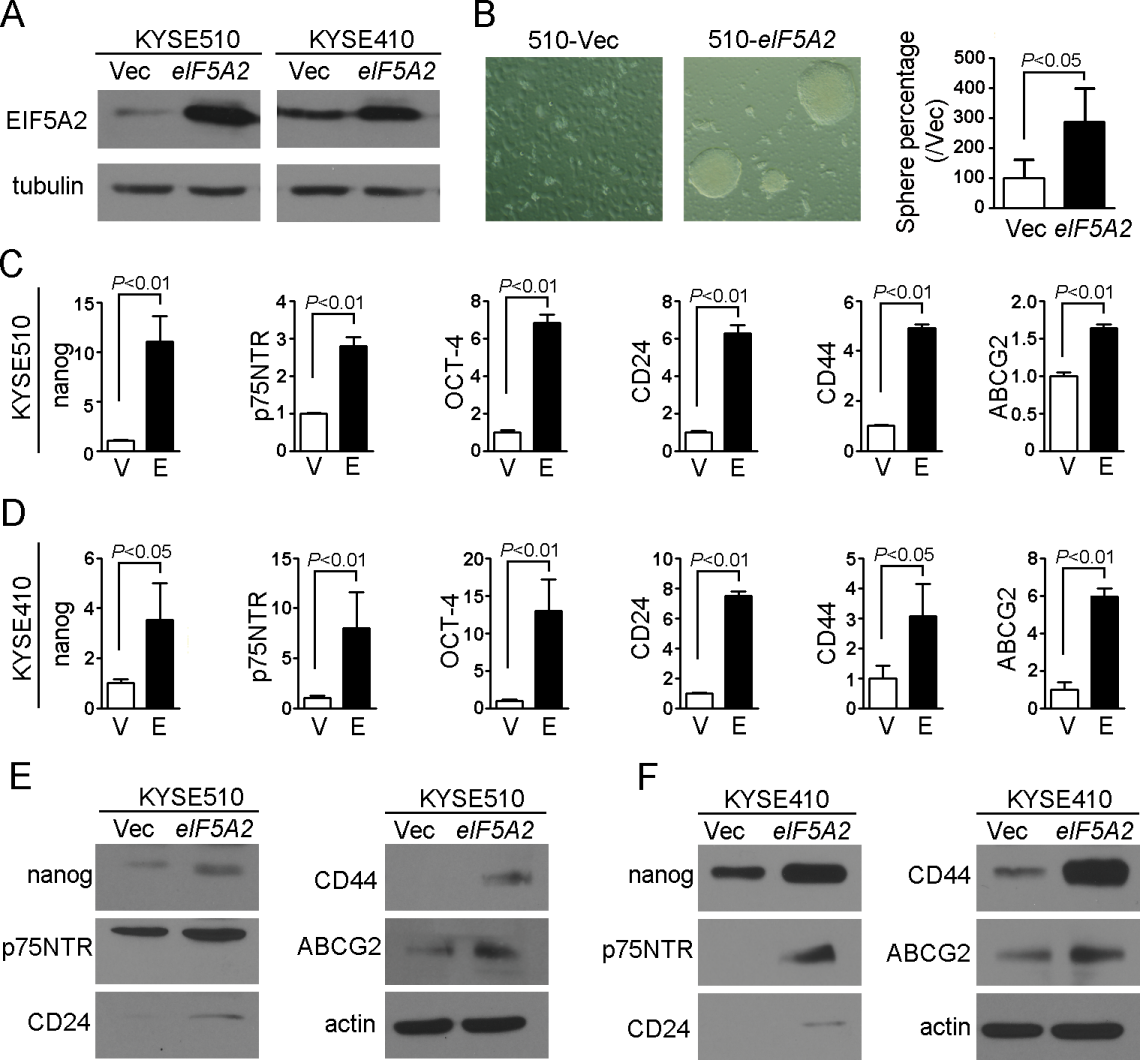
EIF5A2 overexpression increased the stemness of ESCC cells **A.** The protein level was determined in EIF5A2 overexpressed cells (510-*eIF5A2* and 410-*eIF5A2*) and vector control cells (510-Vec and 410-Vec) by western blotting assay. Tubulin was used as loading control. **B.** Representative pictures showing increased sphere-forming ability in 510-*eIF5A2* compared with 510-Vec cells. The results are summarized as the mean ± SEM of three independent assays. **C, D.** Relative expression of stemness-associated genes (*nanog*, *p75NTR* and *OCT-4*), surface antigens associated with ESCC cancer stem cells (*CD24* and *CD44*) and multi drug-resistant transporter gene (*ABCG2*) were compared by qRT-PCR between EIF5A2 overexpressed cells (510-*eIF5A2* and 410-*eIF5A2*) and corresponding vector control cells. **E, F.** The protein levels of nanog, p75NTR, CD24, CD44, ABCG2 increased in EIF5A2 overexpressed cells (510-*eIF5A2* and 410-*eIF5A2*) compared with vector control cells (510-Vec and 410-Vec). Actin was set as loading control.

Our previous study showed that EIF5A2 introduction could increase the tumorigenicity of ESCC cells *in vivo* and *in vitro* [5]. To determine whether *EIF5A2* introduction is sufficient to enhance the stemness of ESCC cells, the *eIF5A2* and vector stably transfected KYSE510 cells were established, and sphere assays were performed. The sphere-forming ability of 510-*eIF5A2* cells was significantly increased (*P* < 0.05) compared to that of vector control cells (510-Vec) (Figure 1A and 1B), suggesting that EIF5A2 increased self-renewal ability.

Then, we extended our analysis by examining the expression levels of stemness-associated genes by qRT-PCR on EIF5A2 overexpressed cells. The RNA levels of *nanog* [10], *p75NTR* [11], *OCT-4* [12], *CD24* [13] and *CD44* [14] increased in the range of 2.8-fold (*p75NTR*) up to 11-fold (*nanog*) in EIF5A2 overexpressed KYSE510 cells (510-*eIF5A2*) compared with vector control cells (*P* < 0.01; Figure 1C). Similar results were also observed in EIF5A2 overexpressed KYSE410 cells (410-*eIF5A2*) (Figure 1A and 1D). Taken together, these findings revealed that EIF5A2 overexpression increased the stemness of ESCC cells. The RNA level of *ABCG2* (ATP-binding cassette, sub-family G, member 2) [15], a multiple drug-resistant transporter gene, also increased by 1.6-fold in 510-*eIF5A2* and 5.9-fold in 410-*eIF5A2* cells (*P* < 0.01; Figure 1C and 1D). The protein levels of nanog, p75NTR, CD24, CD44 and ABCG2 increased in varying degrees in EIF5A2 overexpressed cells (510-*eIF5A2* and 410-*eIF5A2*) compared with their respective vector control cells (Figure 1E and 1F).

### Chemoresistance induced by EIF5A2 overexpression

**Figure 2:**
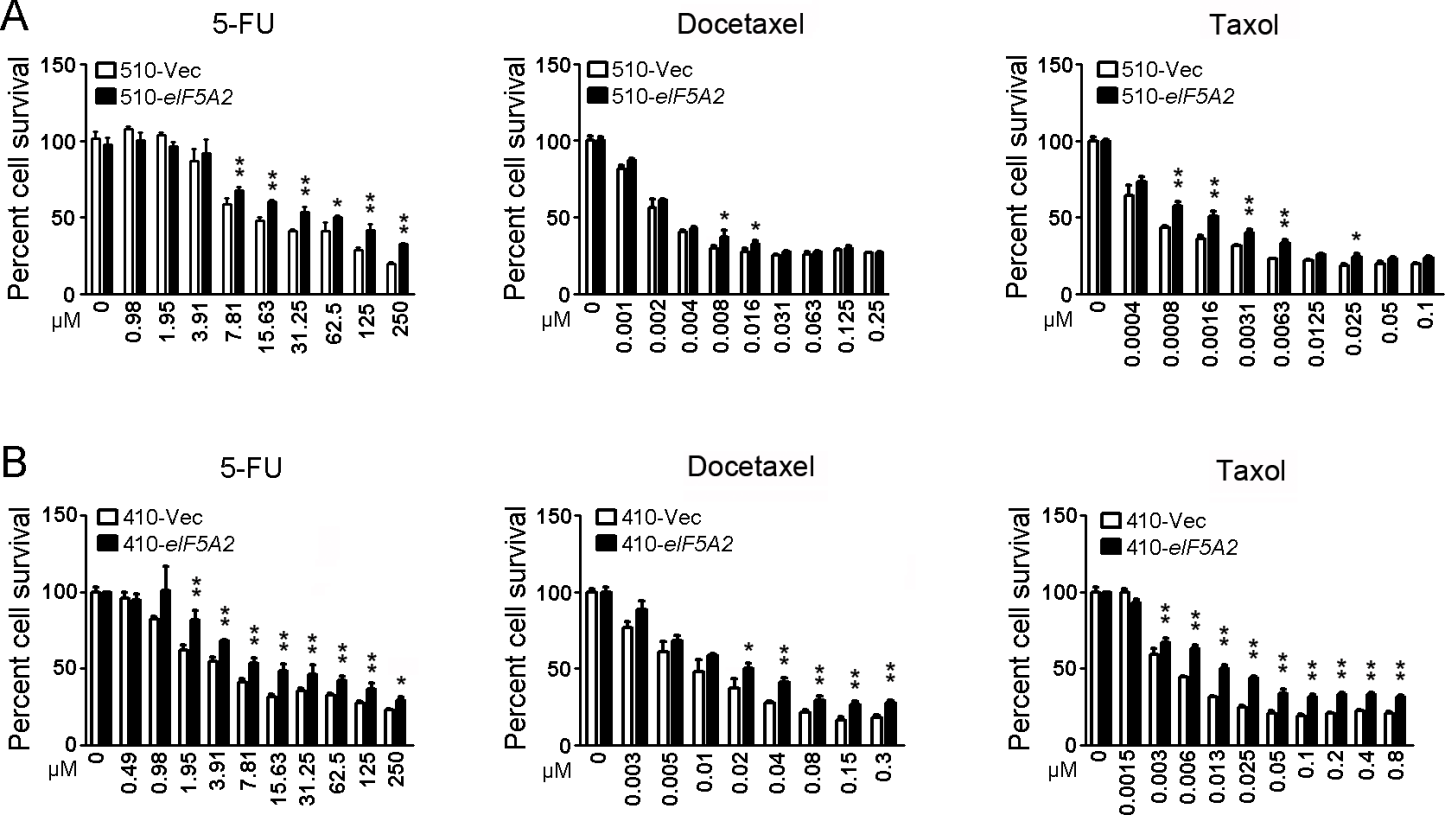
EIF5A2 overexpression induced chemoresistance in ESCC cells **A.** 510-*eIF5A2* cells are more chemoresistant than 510-Vec cells at individual doses of 5-FU, docetaxel and taxol, as shown by XTT assay. *, *P* < 0.05; **, *P* < 0.01. **B.** 410-*eIF5A2* cells are more chemoresistant than 410-Vec cells at individual doses of 5-FU, docetaxel and taxol, as shown by XTT assay. *, *P* < 0.05; **, *P* < 0.01.

Because drug resistance is one of the characters of cancer stem cells and a multiple drug-resistant transporter gene increased in the EIF5A2 overexpressed cells, we next investigated whether EIF5A2 overexpression contributes to the chemoresistance of ESCC cells. For this purpose, XTT assays were used to evaluate the chemoresistance between EIF5A2 overexpressed KYSE510 cells (510-*eIF5A2*) and vector control cells (510-Vec). The 510-*eIF5A2* cells were more resistant to 5-FU than the vector cells (Figure 2A). The results demonstrated that 510-*eIF5A2* cells were more resistant to docetaxel and taxol than 510-Vec cells (Figure 2A). To rule out the possibility that the resistance of chemotherapy induced by EIF5A2 overexpression is specific to KYSE510 and its derivative cells, another ESCC cell line, KYSE410, was next studied. Similar results were observed in the 410-*eIF5A2* cells and 410-Vec cells (Figure 2B).

### Chemo-sensitivity increased by EIF5A2 silencing

**Figure 3:**
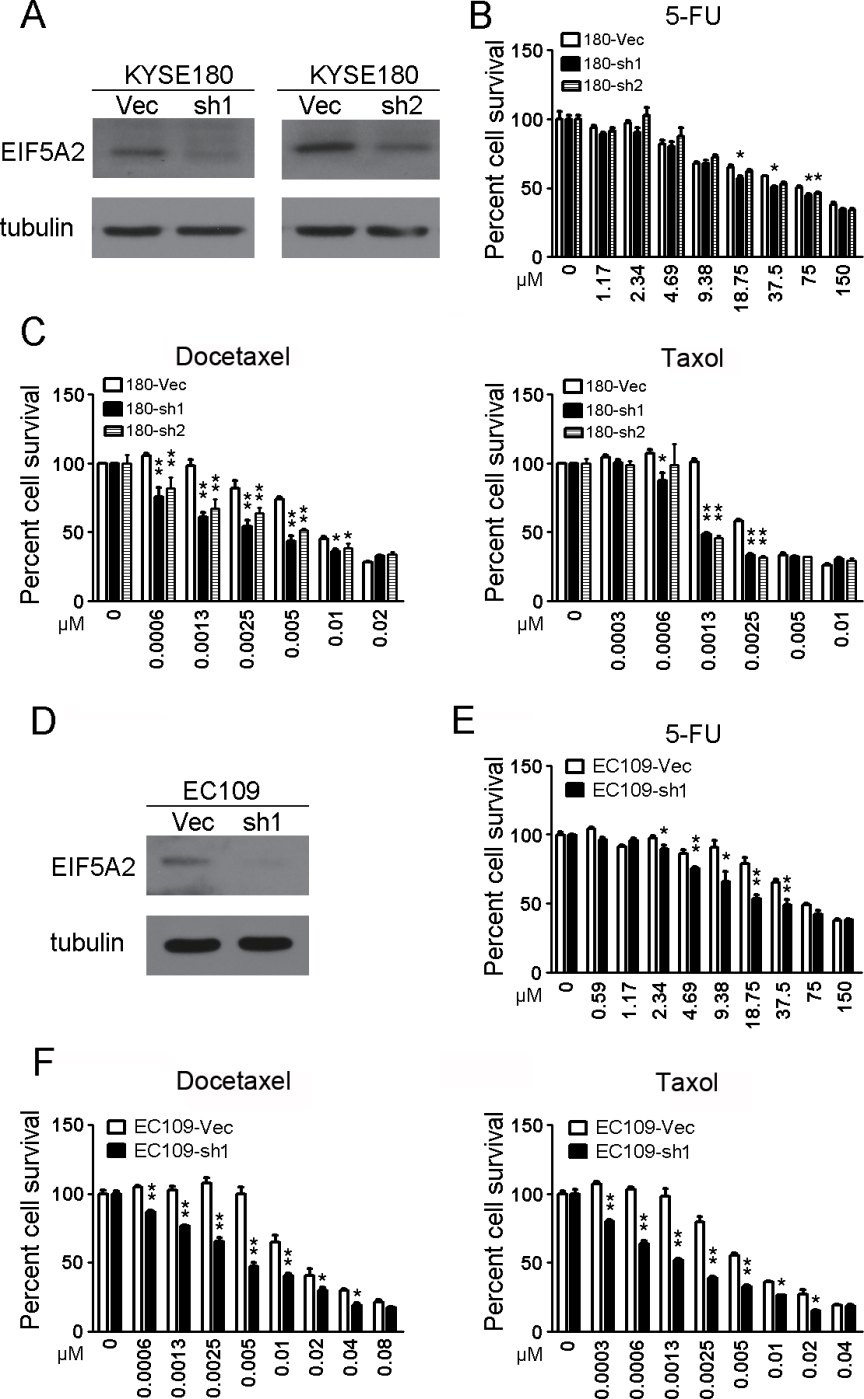
Knock-down of *eIF5A2* increased chemosensitivity **A.** Knock-down of *eIF5A2* by shRNAs (sh1 and sh2) compared with vector control was confirmed in KYSE180 cells by western blotting analysis. Tubulin was set as loading control. **B, C.** The *eIF5A2* knock-down cells (180-sh1 and 180-sh2) were significantly more sensitive than the vector cells at individual 5-FU (B), docetaxel and taxol (C) doses. *, *P* < 0.05; **, *P* < 0.01. **D.** Knock-down of *eIF5A2* was confirmed in EC109 cells by western blotting analysis. Tubulin was set as loading control. **E. F.** The *eIF5A2* knock-down EC109 cells were significantly more sensitive than the vector cells at individual 5-FU (E), docetaxel and taxol (F) doses. *, *P* < 0.05; **, *P* < 0.01.

We next examined whether silencing EIF5A2 expression would increase the chemo-sensitivity by knocking down EIF5A2 expression in KYSE180 and EC109 cells. The ESCC cells were transduced with lentivirus containing shRNAs (sh1 and sh2) against *eIF5A2* (Figure 3A), and cytotoxicity assays were carried out. It was found that KYSE180 with reduced EIF5A2 expression were more sensitive to 5-FU, docetaxel and taxol than the vector control cells (Figure 3B, 3C). Similar results were observed in EC109 cells (Figure 3D, 3E and 3F).

### EIF5A2 conferred chemoresistance by inhibiting apoptosis in ESCC cells

**Figure 4:**
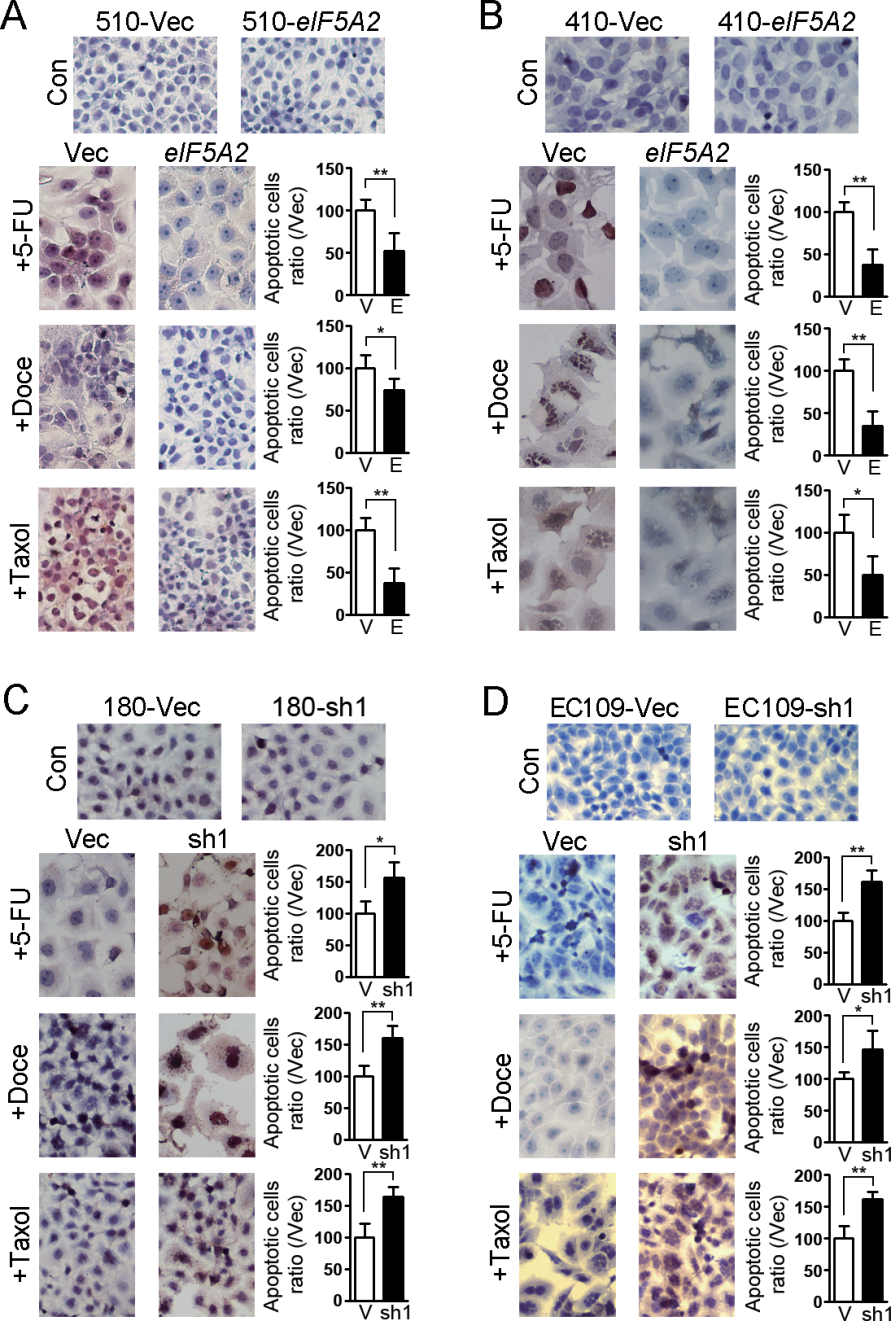
*in situ* cell death assay showed the effect of EIF5A2 on chemoresistance of ESCC cells After treatment with 5-FU, docetaxel and taxol for 48 h, in situ TUNEL assays were performed on cells. Representative images of TUNEL assay are shown. The apoptotic cells were stained a brown color, and the nuclei were counterstained with hematoxylin. EIF5A2 overexpressed 510 **A.** and 410 **B.** cells showed fewer apoptotic cells than their respective vector controls, whereas *eIF5A2* knock-down increased apoptotic cells in 180 **C.** and EC109 cells **D.** compared with their respective vector controls (original magnification: 20×). Summary of apoptotic cells ratio (/Vec) was shown (right). *, *P* < 0.05; **, *P* < 0.01.

**Figure 5:**
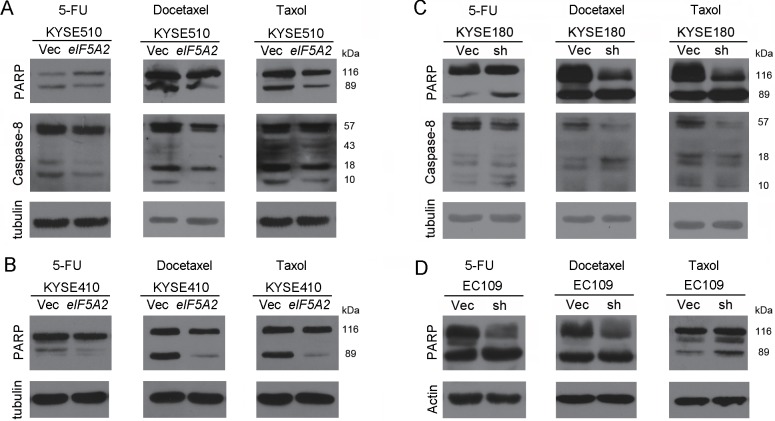
EIF5A2 conferred chemoresistance by inhibiting apoptosis **A.** Cleaved PARP and Caspase-8 were found to be inhibited in *EIF5A2* overexpressed 510 cells in response to 5-FU (50 μM), docetaxel (0.02 μM) and taxol (0.001 μM) when analyzed by western blotting analysis. **B.** Activation of PARP was inhibited in 410-*eIF5A2* cells when treated with 5-FU (5 μM), docetaxol (0.01 μM) and taxol (0.0075 μM) compared with 410-Vec cells. **C.** Cleaved PARP and Caspase-8 were increased in *eIF5A2* knock-down 180 cells treated with 5-FU (60 μM), docetaxel (0.01 μM) and taxol (0.003 μM) compared with 180-Vec cells. **D.** Activation of PARP was increased in EC109-sh cells when treated with 5-FU (50 μM), docetaxel (0.015 μM) and taxol (0.007 μM) compared with vector control cells. Tubulin or actin was set as loading control.

We next elucidated whether EIF5A2 accounts for chemoresistance through inhibiting apoptosis. 510-*eIF5A2* cells and 510-Vec cells were treated with 5-FU, docetaxel and taxol, and *in situ* TUNEL assays were performed. As shown in Figure 4A, the number of apoptotic cells decreased in 510-*eIF5A2* cells compared with 510-Vec cells. Similar results were obtained in KYSE410 derived cells (Figure 4B). In contrast, KYSE180 transduced with shRNA suppressing *eIF5A2* (180-sh1) and vector control cells (180-Vec) were also treated with chemotherapeutic reagents, and the *in situ* TUNEL results also indicated an increased apoptotic response toward 5-FU, docetaxel and taxol in EIF5A2 knock-down cells compared with vector control cells (Figure 4C). Similar results were also obtained in EC109-derived cells (Figure 4D). These findings support that the preferential survival of EIF5A2 overexpressed cells after chemotherapy reagents was attributed to low rates of apoptosis. The activation of PARP and caspase-8, two typical characteristics of cell apoptosis, was also investigated by western blotting in EIF5A2 overexpressed cells and silenced cells. As shown in Figure 5A, the cleaved PARP and caspase-8 were decreased in the 510-*eIF5A2* cells compared with 510-Vec cells after treatment with 5-FU, docetaxel and taxol. In addition, overexpression of *eIF5A2* in KYSE410 significantly decreased the cleaved PARP in comparison with the vector control cells (Figure 5B). As shown in Figure 5C, EIF5A2 knock-down resulted in an increase of PARP and caspase-8 activation in KYSE180 cells after treatment with 5-FU, docetaxel and taxol. Additionally, EIF5A2 knock-down resulted in an activation of PARP in EC109 cells treated with 5-FU, docetaxel and taxol (Figure 5D).

### EIF5A2 overexpression correlated with a poorer outcome of chemotherapy

**Figure 6:**
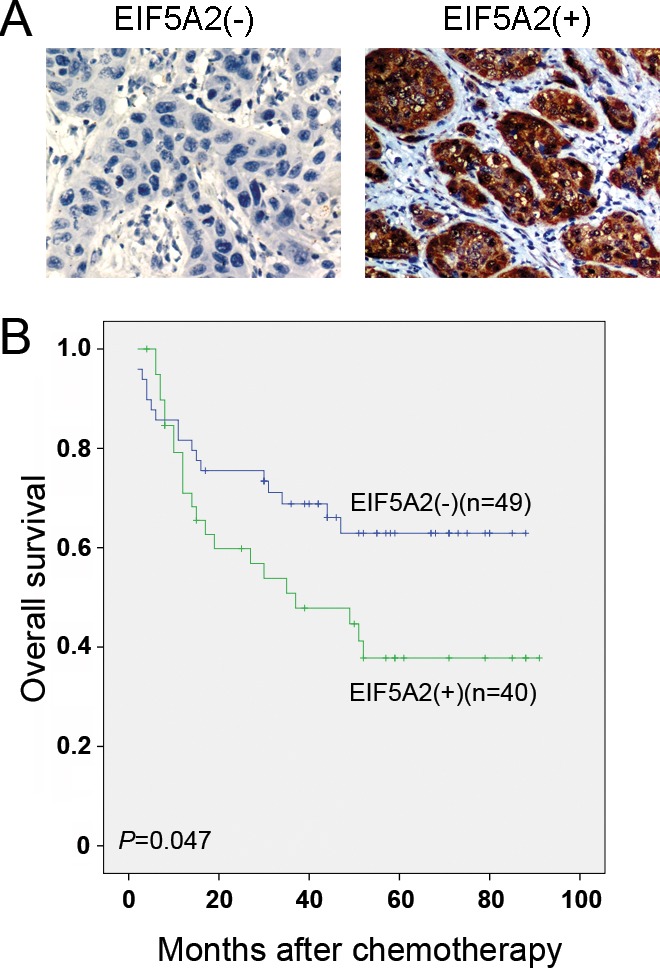
EIF5A2 expression correlated with a poorer outcome of chemotherapy **A.** The representative images of EIF5A2 negative and positive expression in ESCC tissues (original magnification: 20×). **B.** Kaplan-Meier analysis indicated that the overall survival rate of patients with ESCC who received postoperative taxane-based chemotherapy was significantly decreased in the EIF5A2(+) group compared with the EIF5A2(−) group (*P* = 0.047).

To further explore the role of EIF5A2 in chemoresistance in clinical samples, we evaluated the expression of EIF5A2 by IHC in patients who underwent taxane-based chemotherapy after esophagectomy. Positive staining of EIF5A2 was detected in 40 of 89 (44.94%) tumor tissues and negative staining was detected in 49 of 89 (55.06%) tumor cases (Figure 6A). The association study demonstrated that EIF5A2 expression was not significantly associated with gender, age, differentiation status or T status, but it was significantly associated with N status (*P* = 0.011) (Table 1). The Kaplan-Meier survival analysis revealed that EIF5A2 expression was correlated with poorer overall survival (OS) in patients who underwent postoperative taxane-based chemotherapy (log-rank test, *P* = 0.047) (Figure 6B). The mean OS time of the EIF5A2(−) and EIF5A2(+) groups were 62.08 months (95% CI: 52.03–72.14) and 47.77 months (95% CI: 35.97–59.58), respectively.

**Table 1:** Correlation between EIF5A2 expression and clinicopathological features of primary ESCC

Clinicopathological features	All cases	EIF5A2 expression		*P* value
		−	+	
**Gender**				0.924
female	13	7 (53.8%)	6 (46.2%)	
male	76	42 (55.3%)	34 (44.7%)	
**Age**				0.221
< 57 year	53	32 (60.4%)	21 (39.6%)	
≥ 57 year	36	17 (47.2%)	19 (52.8%)	
**Differentiation**				0.184
1–2	62	37 (59.7%)	25 (40.3%)	
3	27	12 (44.4%)	15 (55.6%)	
**T status**				0.119
T1-T2	15	11 (73.3%)	4 (26.7%)	
T3-T4	74	38 (51.4%)	36 (48.6%)	
**N status**				0.011
N0	20	16 (80.0%)	4 (20.0%)	
N1	69	33 (47.8%)	36 (52.2%)	

Note: The Pearson Chi-Square test was used as statistical analysis. Significant difference was defined when *P* < 0.05.

## DISCUSSION

ESCC is one of the most lethal malignancies of the digestive tract in East Asian countries. Often, when many cases are initially diagnosed, the malignancy is already in an advanced stage. Multimodal therapies are necessary to prolong the survival of patients with ESCC. Adjuvant chemotherapy and neo-adjuvant chemotherapy have become the first-line therapy for patients with advanced ESCC [4]. However, the effectiveness of chemotherapy is often limited by drug resistance of the patients. Therefore, it is very important to screen predictive biomarkers for chemosensitivity to select appropriate regimens for patients with ESCC.

Overexpression of EIF5A2 has been detected in many solid tumors, including ovarian cancer [16], hepatocellular carcinoma [17, 18], non-small cell lung cancer [19], bladder cancer [20], melanoma [21], colorectal cancer [22], pancreatic adenocarcinoma [23] and ESCC [5]. It was also reported that EIF5A2 could promote tumor metastasis and angiogenesis in ESCC and hepatocellular carcinoma [5, 6, 18]. Our previous studies found that EMT was involved in the above processes [5, 18]. Because EMT is not only a process to acquire metastasis but also a complicated reprogramming process through which differentiated epithelial cells reverse to an undifferentiated state by acquiring stem cell-like function [24], we tested whether forced expression of EIF5A2 could endow the cells with stem cell properties. Our results indicated that EIF5A2 overexpression enhanced the stemness of the 510-*eIF5A2* cells and induced multiple cancer stem cell markers.

Cancer stem cells are supposed to be responsible for not only tumor initiation and maintenance but also resistance to chemotherapy, radiotherapy and early recurrence. We previously reported that shRNAs suppressing *eIF5A2* combined with chemotherapeutic reagent effectively reduced the volume and weight of xenografts in nude mice [5]. Additionally, some *in vitro* studies reported that knock-down or inhibition of EIF5A2 enhanced the chemosensitivity of HCC cells to 5-FU/doxorubicin and breast cancer cells and bladder cancer cells to doxorubicin [6–9]. Because adjuvant therapy has been increasingly used in the treatment of advanced ESCC, we tested whether EIF5A2 induced chemoresistance of ESCC cells. The overexpression of *eIF5A2* in the ESCC cells (KYSE510 and KYSE410) led to increased chemoresistance to 5-FU, docetaxel and taxol; whereas shRNA-mediated silencing of *eIF5A2* in the ESCC cells (KYSE180 and EC109) increased chemosensitivity towards these chemotherapeutic agents.

Cancer stem cell chemoresistance is explained by slow cycling, efflux of drugs by ATP binding cassette transporters and upregulation of anti-apoptosis [25, 26]. Our study found that the RNA and protein levels of *ABCG2* were increased in EIF5A2 overexpressed cells. ABCG2 is an important member of the ABC transporter superfamily that has been suggested to be involved in multidrug resistance in cancer [27]. It has a diverse range of substrates, including doxorubicin, imatinib, and mitoxantrone [28]. This result is consistent with the previous findings that silencing or inhibition of EIF5A2 led to sensitization of some cancer cells toward doxorubicin [7–9].

5-FU is widely used in cancer chemotherapy. It inhibits thymidylate synthase, an essential enzyme for the *de novo* synthesis of thymidylate and subsequently DNA synthesis. This results in imbalance of dTTP/dUTP and DNA damage. Both the imbalance and DNA damage lead to apoptosis [29]. It was also reported that silencing of endogenous EIF5A2 increased tumor perfusion by remodeling tumor vessels, thus increased the chemosensitivity of HCC cells to 5-FU *in vivo* [6]. In this study, western blotting assay demonstrated that overexpression of EIF5A2 reduced the activation of PARP and caspase-8 after treatment with chemotherapeutics; in contrast, knock-down of EIF5A2 increased the PARP and caspase-8 activation in comparison with the vector control cells. These findings support the role of anti-apoptosis in the gain and manifestation of chemoresistance conferred by EIF5A2 in ESCC cells.

Long-term survival of patients with ESCC is poor due to high rates of therapeutic failure and tumor relapse. Discovering reliable chemosensitivity markers that could accurately predict clinical outcome is beneficial. In our study, the Kaplan-Meier analysis indicated that EIF5A2 expression was associated with a poorer survival in patients with ESCC who underwent taxane-based chemotherapy after esophagectomy. Based on our results, we believe that inhibition of EIF5A2 could be a highly effective strategy in overcoming 5-FU or taxane-based chemoresistance in a clinical setting. N1-guanyl-1, 7-diaminoheptane (GC7), which inhibits EIF5A2 activation [8], could be considered as a combination therapy to enhance chemosensitivity in patients with ESCC.

## MATERIALS AND METHODS

### Patients and tissue samples

Between December 2004 and August 2009, a retrospective cohort of 89 patients with ESCC who underwent esophagectomy with adjuvant chemotherapy at Sun Yat-sen University Cancer Center was enrolled in this study. The patients were diagnosed with primary ESCC, and none of them had received preoperative chemotherapy, preoperative radiotherapy, or concurrent chemoradiotherapy. Staging was uniformly defined using the criteria set forth by the 6^th^ edition of American Joint Committee on Cancer Guidelines. Of the 89 patients who received taxane-based chemotherapy, 62 (69.7%) underwent taxol-based regimens and 27 (30.3%) underwent docetaxel-based regimens. The age of the patients ranged from 35 to 72 years at the time of surgery (median age: 55 years). The median follow-up time was 60 months (inverted Kaplan-Meier analysis) [30]. Tissue samples used in the study were approved by the Committees for Ethical Review of Research Involving Human Subjects in Sun Yat-sen University Cancer Center.

### Cell lines

ESCC cell lines KYSE510, KYSE180, and KYSE410 were obtained from DSMZ, the German Resource Center for Biological material [31]. EC109 was kindly provided by Professor G. Srivastava [32]. Cells were maintained in DMEM medium (Gibco BRL, NY) supplemented with 10% fetal bovine serum (ExCell, Shanghai, China). 293FT cells were purchased from Invitrogen (Carlsbad, CA).

### Virus production and stable cell lines established

The plenti-*eIF5A2* construct was purchased from GeneCopoeia (Guangzhou, China). The constructs containing shRNAs suppressing *eIF5A2* were from Sigma-Aldrich company (Shanghai, China). The lentiviruses were packaged in 293FT cells according to the manufacturer's protocol (Invitrogen, CA). The stable cell lines (overexpressed or knock-down) were established after cells were transduced with lentiviruses (plenti-*eIF5A2* or shRNAs) and selected with puromycin. The concentration of puromycin for the cell lines selection was listed as: KYSE510 (1.2 μg/ml), KYSE410 (0.6 μg/ml), KYSE180 (0.5 μg/ml) and EC109 (0.6 μg/ml).

### Sphere assay

510-*eIF5A2* and vector control cells were seeded into 24-well polyHEMA-coated plates (Sigma-Aldrich, St Louis, MO) and cultured for 2 weeks in DMEM/F12 medium supplemented with 4 μg/mL insulin (Bioind, Kibbutz Beit haemek, Israel), B27 (Gibco, Grand island, NY), 20 ng/mL EGF (PeproTech, Rocky Hill, NJ) and 10 ng/mL basic FGF (PeproTech). Cells were replenished with fresh medium every second day [33]. The spheres in the suspension culture were counted under microscope (Olympus IX71) [34].

### Quantitative real-time PCR (qRT-PCR)

Total RNA from cells was extracted using Trizol (Invitrogen, CA) according to the manufacturer's instructions. Reverse transcription was performed using the SuperScript reverse transcriptase (Invitrogen, CA). The cDNA was subjected to qRT-PCR using the SYBR Green reagent (Roche, Mannheim, Germany) and an ABI 7900HT Fast Real-time PCR system (Applied Biosystems). 18S rRNA was used as internal control. The assays were performed in triplicate, and values were normalized using the internal control. PCR products were subjected to dissociation curve analysis to exclude amplification of nonspecific products. Quantitative data were analyzed using the ΔCt method. The primer sequences are listed in the Supplementary Data.

### Western blotting

Cells were lysed with RIPA buffer (Cell signaling technology, Danvers, MA), the proteins were fractionated by SDS-PAGE and western blotting was performed. The blots were incubated with diluted primary antibodies overnight at 4°C. Then, blots were exposed to the appropriate peroxidase-conjugated secondary antibodies (Cell Signaling Technology, MA) at 1:3000 for 2 h at room temperature. Finally blots were developed with Luminata Crescendo western HRP substrate (Millipore, MA). The following antibodies were used: EIF5A2 (1:1000) (Wolwo, Shanghai, China), Casepase-8 (1:1000), PARP (1:1000), CD44 (1:500), nanog (1:500) and p75NTR (1:500) (Cell Signaling Technology, MA), ABCG2 (1:500), CD24 (1:500), β-tubulin (1:1000) and actin (1:1000) (Santa Cruz Biotechnology, TX).

### Cytotoxicity assay

The drugs used in the study were: 5-FU (SunRise Ltd., Shanghai, China), docetaxel (Aventis Pharma S.A., Bridgewater, NJ) and taxol (Wellso Pharma, Beijing, China). Chemotherapy-induced cytotoxicity was determined using an XTT assay (CCK-8, Dojindo, Kyushu, Japan). Cells were plated in 96-well plates at the appropriate density. Twenty-four hours later, cells were treated with 2-fold diluted concentrations of drugs for another 70 hours at 37°C. Then the XTT assay was performed and assayed at OD450 according to the manufacturer's instructions. Three independent assays were performed.

### *In situ* cell death detection assay

Apoptotic cells were detected using an *in situ* Cell Death Detection Kit (Roche Applied Science, IN) according to the protocol. Cells were plated on the cover slides and treated with different chemotherapy regents. Forty-eight hours later, cells were washed with PBS and fixed in 4% paraformaldehyde. After blocking and incubation in permeabilisation buffer, apoptotic cells were determined by the terminal deoxyribonucleotidyl transferase (TdT)-mediated dUTP-digoxigenin nick end labeling (TUNEL) assay. Signal was converted by adding Concerter-POD and analyzed under light microscope.

### Immunohistochemistry (IHC)

IHC was performed using the standard streptavidin-biotin-peroxidase complex method. Briefly, the tissue sections were deparaffinized and subjected to antigen retrieval in EDTA antigen retrieval buffer (ZSGB-BIO, Beijing, China). Then the sections were incubated with the diluted primary antibody EIF5A2 (1:50) (Wolwo, Shanghai, China) overnight at 4°C. After incubation with horseradish peroxidase-linked secondary antibody (Real Envision Detection Kit, Gene Tech, Shanghai, China) for 30 min, the sections were counterstained with Mayer's hematoxylin. Expression of EIF5A2 was scored as absent (total absence of staining), very weak (faint staining in < 25% of tumor cells), moderate (moderate staining in ≥ 25% to < 75%, or strong staining in < 25% of tumor cells), and strong (moderate staining in ≥ 75% or strong staining in ≥ 25% of tumor cells). In the present study, absent/very weak staining was defined as negative staining and moderate/strong staining as positive staining.

### Statistical analysis

Statistical calculations were carried out with the SPSS 16.0 software package (SPSS, Inc., Chicago, IL). Survival curves were generated according to the Kaplan-Meier method and log-rank tests. All data were expressed as the means ± SEM. The results were considered statistically significant when *P* < 0.05.

## SUPPLEMENTARY TABLE



## Abbreviations

eIF5A2: eukaryotic translation initiation factor 5A2
ESCC: esophageal squamous cell carcinoma
EMT: epithelial-mesenchymal transition
5-FU: 5-fluorouracil
qRT-PCR: quantitative real-time polymerase chain reaction
TUNEL: (TdT)-mediated dUTP-digoxigenin nick end labeling
IHC: immunohistochemistry
shRNA: short hairpin RNA
OS: overall survival

## References

[1] Ferlay J, Shin HR, Bray F, Forman D, Mathers C, Parkin DM (2010). Estimates of worldwide burden of cancer in 2008: GLOBOCAN 2008. International journal of cancer Journal international du cancer.

[2] Ando N, Iizuka T, Ide H, Ishida K, Shinoda M, Nishimaki T, Takiyama W, Watanabe H, Isono K, Aoyama N (2003). Surgery plus chemotherapy compared with surgery alone for localized squamous cell carcinoma of the thoracic esophagus: a Japan Clinical Oncology Group Study—JCOG9204. Journal of clinical oncology: official journal of the American Society of Clinical Oncology.

[3] Iinuma H, Fukushima R, Inaba T, Tamura J, Inoue T, Ogawa E, Horikawa M, Ikeda Y, Matsutani N, Takeda K (2014). Phase I clinical study of multiple epitope peptide vaccine combined with chemoradiation therapy in esophageal cancer patients. Journal of translational medicine.

[4] Lyu X, Huang J, Mao Y, Liu Y, Feng Q, Shao K, Gao S, Jiang Y, Wang J, He J (2014). Adjuvant chemotherapy after esophagectomy: is there a role in the treatment of the lymph node positive thoracic esophageal squamous cell carcinoma?. Journal of surgical oncology.

[5] Li Y, Fu L, Li JB, Qin Y, Zeng TT, Zhou J, Zeng ZL, Chen J, Cao TT, Ban X (2014). Increased expression of EIF5A2, via hypoxia or gene amplification, contributes to metastasis and angiogenesis of esophageal squamous cell carcinoma. Gastroenterology.

[6] Wang FW, Cai MY, Mai SJ, Chen JW, Bai HY, Li Y, Liao YJ, Li CP, Tian XP, Kung HF (2014). Ablation of EIFA2 induces tumor vasculature remodeling and improves tumor response to chemotherapy via regulation of matrix metalloproteinase 2 expression. Oncotarget.

[7] Lou B, Fan J, Wang K, Chen W, Zhou X, Zhang J, Lin S, Lv F, Chen Y (2013). N1-guanyl-1, 7-diaminoheptane (GC7) enhances the therapeutic efficacy of doxorubicin by inhibiting activation of eukaryotic translation initiation factor 5A2 (eIF5A2) and preventing the epithelial-mesenchymal transition in hepatocellular carcinoma cells. Experimental cell research.

[8] Yang J, Yu H, Shen M, Wei W, Xia L, Zhao P (2014). N1-guanyl-1, 7-diaminoheptane sensitizes bladder cancer cells to doxorubicin by preventing epithelial-mesenchymal transition through inhibition of eukaryotic translation initiation factor 5A2 activation. Cancer science.

[9] Liu Y, Du F, Chen W, Yao M, Lv K, Fu P (2014). EIF5A2 is a novel chemoresistance gene in breast cancer. Breast cancer.

[10] Hwang CC, Nieh S, Lai CH, Tsai CS, Chang LC, Hua CC, Chi WY, Chien HP, Wang CW, Chan SC (2014). A retrospective review of the prognostic value of ALDH-1, Bmi-1 and Nanog stem cell markers in esophageal squamous cell carcinoma. PloS one.

[11] Okumura T, Tsunoda S, Mori Y, Ito T, Kikuchi K, Wang TC, Yasumoto S, Shimada Y (2006). The biological role of the low-affinity p75 neurotrophin receptor in esophageal squamous cell carcinoma. Clinical cancer research: an official journal of the American Association for Cancer Research.

[12] Shahryari A, Rafiee MR, Fouani Y, Oliae NA, Samaei NM, Shafiee M, Semnani S, Vasei M, Mowla SJ (2014). Two novel splice variants of SOX2OT, SOX2OT-S1, and SOX2OT-S2 are coupregulated with SOX2 and OCT4 in esophageal squamous cell carcinoma. Stem cells.

[13] Yang CH, Wang HL, Lin YS, Kumar KP, Lin HC, Chang CJ, Lu CC, Huang TT, Martel J, Ojcius DM (2014). Identification of CD24 as a cancer stem cell marker in human nasopharyngeal carcinoma. PloS one.

[14] Zhao JS, Li WJ, Ge D, Zhang PJ, Li JJ, Lu CL, Ji XD, Guan DX, Gao H, Xu LY (2011). Tumor initiating cells in esophageal squamous cell carcinomas express high levels of CD44. PloS one.

[15] Tsunoda S, Okumura T, Ito T, Kondo K, Ortiz C, Tanaka E, Watanabe G, Itami A, Sakai Y, Shimada Y (2006). ABCG2 expression is an independent unfavorable prognostic factor in esophageal squamous cell carcinoma. Oncology.

[16] Guan XY, Sham JS, Tang TC, Fang Y, Huo KK, Yang JM (2001). Isolation of a novel candidate oncogene within a frequently amplified region at 3q26 in ovarian cancer. Cancer research.

[17] Lee NP, Tsang FH, Shek FH, Mao M, Dai H, Zhang C, Dong S, Guan XY, Poon RT, Luk JM (2010). Prognostic significance and therapeutic potential of eukaryotic translation initiation factor 5A (eIF5A) in hepatocellular carcinoma. International journal of cancer Journal international du cancer.

[18] Tang DJ, Dong SS, Ma NF, Xie D, Chen L, Fu L, Lau SH, Li Y, Li Y, Guan XY (2010). Overexpression of eukaryotic initiation factor 5A2 enhances cell motility and promotes tumor metastasis in hepatocellular carcinoma. Hepatology.

[19] He LR, Zhao HY, Li BK, Liu YH, Liu MZ, Guan XY, Bian XW, Zeng YX, Xie D (2011). Overexpression of eIF5A-2 is an adverse prognostic marker of survival in stage I non-small cell lung cancer patients. International journal of cancer Journal international du cancer.

[20] Wei JH, Cao JZ, Zhang D, Liao B, Zhong WM, Lu J, Zhao HW, Zhang JX, Tong ZT, Fan S (2014). EIF5A2 predicts outcome in localised invasive bladder cancer and promotes bladder cancer cell aggressiveness *in vitro* and *in vivo*. British journal of cancer.

[21] Khosravi S, Wong RP, Ardekani GS, Zhang G, Martinka M, Ong CJ, Li G (2014). Role of EIF5A2, a downstream target of Akt, in promoting melanoma cell invasion. British journal of cancer.

[22] Zhu W, Cai MY, Tong ZT, Dong SS, Mai SJ, Liao YJ, Bian XW, Lin MC, Kung HF, Zeng YX (2012). Overexpression of EIF5A2 promotes colorectal carcinoma cell aggressiveness by upregulating MTA1 through C-myc to induce epithelial-mesenchymaltransition. Gut.

[23] Wei YX, Chen G, You L, Zhao YP (2013). Expression of eukaryotic translation initiation factor 5A2 in pancreatic adenocarcinoma and its correlation with the prognosis. Zhongguo yi xue ke xue yuan xue bao Acta Academiae Medicinae Sinicae.

[24] Li L, Li W (2015). Epithelial-mesenchymal transition in human cancer: Comprehensive reprogramming of metabolism, epigenetics, and differentiation. Pharmacol Ther.

[25] Eyler CE, Rich JN (2008). Survival of the fittest: cancer stem cells in therapeutic resistance and angiogenesis. Journal of clinical oncology: official journal of the American Society of Clinical Oncology.

[26] Abdullah LN, Chow EK (2013). Mechanisms of chemoresistance in cancer stem cells. Clinical and translational medicine.

[27] Robey RW, To KK, Polgar O, Dohse M, Fetsch P, Dean M, Bates SE (2009). ABCG2: a perspective. Advanced drug delivery reviews.

[28] Stacy AE, Jansson PJ, Richardson DR (2013). Molecular pharmacology of ABCG2 and its role in chemoresistance. Molecular pharmacology.

[29] Van Triest B, Pinedo HM, Giaccone G, Peters GJ (2000). Downstream molecular determinants of response to 5-fluorouracil and antifolate thymidylate synthase inhibitors. Annals of oncology: official journal of the European Society for Medical Oncology / ESMO.

[30] Schemper M, Smith TL (1996). A note on quantifying follow-up in studies of failure time. Controlled clinical trials.

[31] Shimada Y, Imamura M, Wagata T, Yamaguchi N, Tobe T (1992). Characterization of 21 newly established esophageal cancer cell lines. Cancer.

[32] Wong ML, Tao Q, Fu L, Wong KY, Qiu GH, Law FB, Tin PC, Cheung WL, Lee PY, Tang JC (2006). Aberrant promoter hypermethylation and silencing of the critical 3p21 tumour suppressor gene, RASSF1A, in Chinese oesophageal squamous cell carcinoma. International journal of oncology.

[33] Liu L, Dai Y, Chen J, Zeng T, Li Y, Chen L, Zhu YH, Li J, Li Y, Ma S (2014). Maelstrom promotes hepatocellular carcinoma metastasis by inducing epithelial-mesenchymal transition by way of Akt/GSK-3beta/Snail signaling. Hepatology.

[34] Chiu WT, Huang YF, Tsai HY, Chen CC, Chang CH, Huang SC, Hsu KF, Chou CY (2015). FOXM1 confers to epithelial-mesenchymal transition, stemness and chemoresistance in epithelial ovarian carcinoma cells. Oncotarget.

