# Supplementary material for: Stemness and chemotherapeutic drug resistance induced by EIF5A2 overexpression in esophageal squamous cell carcinoma
Source: Oncotarget. 2015 Jul 20;6(28):26079–89. doi: 10.18632/oncotarget.4581 (PMC4694887; doi:10.18632/oncotarget.4581)
Supplement: Supplementary file 1 [file oncotarget-06-26079-s001.pdf]

SUPPLEMENTARY TABLE

Supplementary Table S1: Primer sequences

OCT4-F1	5′ CTTGAATCCCGAATGGAAAGGG 3′
OCT4-R1	5′ GTGTATATCCCAGGGTGATCCTC 3′
CD24-F1	5′ CTCCTACCCACGCAGATTTATTC 3′
CD24-R1	5′ AGAGTGAGACCACGAAGAGAC 3′
Nanog-F1	5′ TTTGTGGGCCTGAAGAAAACT 3′
Nanog-R1	5′ AGGGCTGTCCTGAATAAGCAG 3′
p75NTR-F1	5′ CCTACGGCTACTACCAGGATG 3′
p75NTR-R1	5′ CACACGGTGTTCTGCTTGT 3′
CD44-F1	5′ CTGCCGCTTTGCAGGTGTA 3′
CD44-R1	5′ CATTGTGGGCAAGGTGCTATT 3′
ABCG2-F1	5′ ACGAACGGATTAACAGGGTCA 3′
ABCG2-R1	5′ CTCCAGACACACCACGGAT 3′
18S-F1	5′ CTCTTAGCTGAGTGTCCCGC 3′
18S-R1	5′ CTGATCGTCTTCGAACCTCC 3′
